# Wild black bears harbor simple gut microbial communities with little difference between the jejunum and colon

**DOI:** 10.1038/s41598-020-77282-w

**Published:** 2020-11-27

**Authors:** Sierra J. Gillman, Erin A. McKenney, Diana J. R. Lafferty

**Affiliations:** 1 Wildlife Ecology and Conservation Science Lab, Department of Biology, Northern Michigan University, Marquette, MI 49855 USA; 2 Department of Applied Ecology, North Carolina State University, Raleigh, NC 27607 USA

**Keywords:** Ecology, Conservation biology, Evolutionary ecology, Microbial ecology

## Abstract

The gut microbiome (GMB), comprising the commensal microbial communities located in the gastrointestinal tract, has co-evolved in mammals to perform countless micro-ecosystem services to facilitate physiological functions. Because of the complex inter-relationship between mammals and their gut microbes, the number of studies addressing the role of the GMB on mammalian health is almost exclusively limited to human studies and model organisms. Furthermore, much of our knowledge of wildlife–GMB relationships is based on studies of colonic GMB communities derived from the feces of captive specimens, leaving our understanding of the GMB in wildlife limited. To better understand wildlife–GMB relationships, we engaged hunters as citizen scientists to collect biological samples from legally harvested black bears (*Ursus americanus*) and used 16S rRNA gene amplicon sequencing to characterize wild black bear GMB communities in the colon and jejunum, two functionally distinct regions of the gastrointestinal tract. We determined that the jejunum and colon of black bears do not harbor significantly different GMB communities: both gastrointestinal sites were dominated by Firmicutes and Proteobacteria. However, a number of bacteria were differentially enriched in each site, with the colon harboring twice as many enriched taxa, primarily from closely related lineages.

## Introduction

Over the last century, humankind has appreciably altered Earth’s ecosystems in myriad ways. Anthropogenic changes such as over-exploitation of natural resources, urbanization, pollution, and human-mediated climate change have caused irrevocable biodiversity loss, with large carnivores suffering the greatest population declines and range contractions of all terrestrial mammals worldwide^1^. For example, six of the eight extant bear species are considered vulnerable to extinction. Whether it be giant pandas (*Ailuropoda melanoleuca*) suffering from severe habitat fragmentation^2^, or polar bears (*Ursus maritimus*) facing reduced foraging opportunities due to rapidly retreating summer sea ice^3^, or Asiatic black bears (*Ursus thibetanus*) subject to intensified poaching for commercial trade of bear body parts (e.g., bear bile)^4^, bears are particularly poignant examples of the hurdles ecologists and conservationists face in the struggle to save ecologically and culturally important carnivores around the world.

While the threats carnivores face in their degraded external environments are relatively well-known (e.g., depleted prey, genetic isolation), the threats carnivores face from changes in their own internal environments, as a consequence of human-mediated environmental perturbations, are almost entirely unknown. For example, how do changes in carnivore gut microbiome (GMB), the consortia of microorganisms (i.e., bacteria, fungi, viruses) within the gastrointestinal tract, affect host fitness? In fact, the intimate co-evolution between vertebrate hosts and their GMB is an emerging area of interest in wildlife ecology. Previous studies have demonstrated that GMBs perform countless micro-ecosystem services for their hosts^5^, facilitating critical physiological processes such as digestion^6^ and vitamin synthesis^7^, immune system maintenance^8^ as well as host weight regulation^9^. Indeed, research provides strong evidence of intraspecific variation and heritability in GMBs, suggesting that GMBs may affect host phenotype and ultimately hosts’ adaptive potential (see review by Hauffe and Barelli^10^).

Though intrinsic and extrinsic factors influencing GMBs are multifaceted (e.g., host’s external environment, sex, life stage, phylogeny, and diet)^11–14^, human-mediated shifts in GMB community composition may lead to altered micro-ecosystem function, affecting nutrient uptake and host health^15,16^. Specifically, as GMB composition is dependent particularly upon the host’s habitat and consequently food availability and therefore diet, the GMB may act as a mechanism for plasticity, enabling hosts to acclimate to a changing environment brought about by anthropogenic pressures and resulting in long-term implications for host survival and evolution. Further, due to the sensitivity of the GMB to host habitat, the composition and functional profile of wildlife–GMBs could serve as a proxy for habitat quality and could therefore provide a valuable tool in wildlife management and monitoring initiatives^13^.

Much of our knowledge of wildlife–GMB relationships is based on studies of captive specimens, due in part to the elusive nature and limited knowledge of dietary intake of many wild species. However, recent studies have revealed that captivity can alter GMB community composition^17–19^. Further, most wildlife–GMB research has focused on colon samples (i.e., feces), where fiber fermentation occurs, understandably because collecting samples from other regions of the gastrointestinal tract is highly invasive. However, within omnivores and carnivores nearly 90% of fats, carbohydrates, and proteins are absorbed in the jejunum, the middle section of the small intestine^20,21^. Thus, by focusing research attention on microbial communities associated with the colon, scientists are unable to fully understand important evolutionary relationships between wildlife and their GMBs in other functionally distinct regions of the gastrointestinal tract–relationships that may be important when considering wildlife management and conservation initiatives. Incorporating analyses of GMB community composition and structure into ecological research initiatives may aid our understanding of wildlife–GMB co-evolution and provide novel insights into host health (see review by Amato^22^).

The American black bear (*Ursus americanus*) is both an ecologically and culturally important large carnivore with a unique life history. Black bears have retained a physiologically simple gastrointestinal tract characteristic of carnivores (i.e., lack of cecum, short gastrointestinal tract length)^23^, but consume an omnivorous diet and exhibit substantial among-individual dietary variation^24^ and behavioral plasticity^25,26^. Beyond serving as both predators and prey, black bears function as important seed dispersers^27^ due to their extremely rapid digestion time^28^. Black bears also undergo four distinct physiological stages, the most complex annual cycle of all carnivores^29^. In winter months, black bears undergo torpor (i.e., Stage I), characterized by fasting and inactivity when food resources are scarce. In early spring, black bears enter walking hibernation (i.e., Stage II), an anorectic phase during which black bears do not eat for the initial 10 to 14 days after den emergence. Black bears then resume normal activity (i.e., Stage III), eating and drinking at will. During fall months, black bears enter hyperphagia (i.e., Stage IV), a period of increased caloric intake mediated by changes in seasonal digestive ability^30^ during which they can gain up to 1 kg daily^31^, which is essential for surviving Stage I. While the length of torpor varies by latitude and weather conditions, torpor can last up to seven months^32^, during which time black bears typically do not eat. Additionally, black bears have low reproductive rates due to slow maturation rate, long-term maternal care, and relatively small litters (two to four cubs) that depend on maternal fat reserves acquired during hyperphagia^33^. Moreover, although black bears are one of two bear species listed as least concern on the IUCN Red List of Threatened Species^34^, black bear geographic range is increasingly fragmented due to urbanization across North America. With diminishing habitat, black bears live in closer proximity to humans, resulting in increased human–wildlife conflict. Furthermore, thousands of black bears are legally harvested each year in the USA and Canada, providing an opportunity to train hunters as citizen scientists to collect biological samples from regions of the gastrointestinal tract that would otherwise require invasive collection. Thus, as a common carnivore with a broad diet and populations widely distributed and harvested across much of North America, black bears are an excellent model for investigating carnivore–GMB relationships.

In the current study, we aimed to characterize and compare black bear GMB communities associated with two functionally distinct regions of the gastrointestinal tract, the jejunum and colon. Based on previous GMB research on omnivores^35–37^, we hypothesized that the jejunum and colon would harbor distinct GMB community structures (e.g., evenness, richness, phylogenetics). Additionally, as hunters in Michigan can legally harvest any bear, excluding cubs (i.e., individuals < 42 in. from nose to tail) and sows with cubs, the current data set includes samples from individuals across different age-classes. Previous studies have revealed distinct GMB communities in cubs versus adult giant pandas^38^ and across age-classes in hyenas^39^, whereas McKenney et al.^12^ demonstrated that GMB community composition shifts with age from birth to weaning in captive lemurs, with GMBs growing more complex but more similar, stable climax communities. We therefore predicted that black bears belonging to different age-classes would harbor distinct microbiomes, and that alpha diversity would increase but beta diversity would decrease with age. To our knowledge, our research is the first investigation of GMB associated with operationally distinct regions of the gastrointestinal tract in a wild carnivore population.

## Results

After quality filtering and rarefaction to 1050 sequences/sample, categorizing black bears into three age-classes: yearlings (1 year old), subadults (2–3 years old), and adults (≥ 4 years old), and removing samples with no known age assigned (*n* = 4), we retained 61 of 66 samples for downstream statistical analysis.

### Alpha and beta diversity

Overall, the top linear mixed effect model (LMM) for Faith’s phylogenetic diversity (here after Faith’s PD) included no interactions (Supplementary Table S1) and only age-class significantly influenced Faith’s PD (β_sub_ = 0.3, β_adu_ = −0.07, *χ*^2^ = 8.62, *p* = 0.014; Table 1); for means of each alpha diversity index, see Supplementary Figure S1 and Supplementary Table S2. Contrasts of estimated marginal means (EMMs) for age-class revealed that Faith’s PD was driven by differences between subadults and adults only (*p* = 0.05; Table 2). We found no significant differences in either Shannon diversity or inverse Simpson diversity between gastrointestinal tract sites, sexes, or among age-classes in the top LMM models (Table 1).

**Table 1:** Wald's *χ*^2^ results for α-diversity on top LMM model performed on alpha diversity indices.

Effect	*χ*^2^	df	*p*
A. Faith's PD: R^2^_c_ = 0.214, R^2^_m_ =0 .141			
GIT	2.18	1	0.14
Sex	0.09	1	0.76
Age class	7.17	2	0.03
B. Shannon diversity: R^2^_c_ = 0.041, R^2^_m_ = 0.025			
GIT	0.13	1	0.72
Sex	0.06	1	0.8
Age class	1.36	2	0.51
C. Inverse Simpson diversity: R^2^_c_ = 0.025, R^2^_m_ = 0.017			
GIT	0.0002	1	0.99
Sex	0.12	1	0.67
Age class	0.84	2	0.66

**Table 2:** Emmeans for LMM models of Faith's PD comparing black bear age-classes.

Contrast	Estimate	SE	df	t-ratio	*p*
Age class p value adjustment: Tukey method for comparing a of three estimates					
Yearling–Subadult	− 0.3	0.17	27.7	− 1.73	0.21
Yearling–Adult	0.07	0.18	28	0.39	0.92
Subadult–Adult	0.37	0.15	28.1	2.52	0.05

Degrees-of-freedom: Satterthwaite.

Confidence level: 0.95

**Figure 1:**
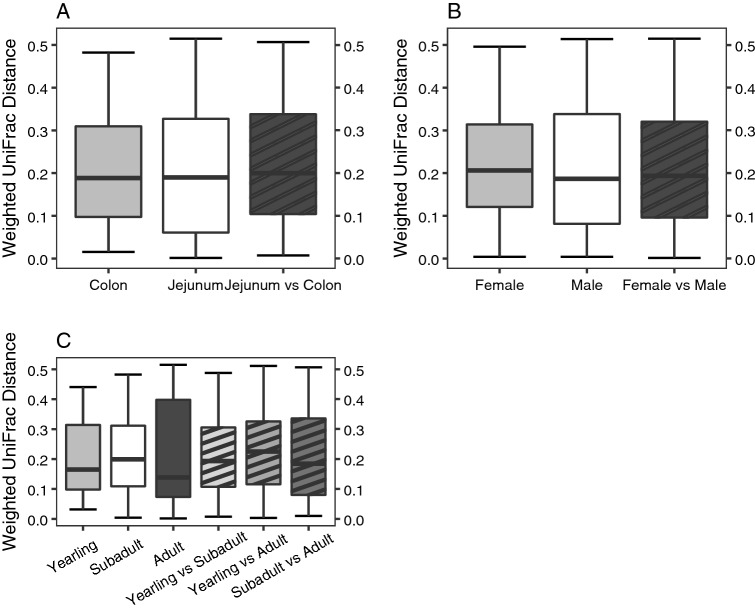
Boxplots of weighted UniFrac reveal less GMB variation between (**A**) gastrointestinal sites and (**C**) sexes, whereas (**C**) age-classes show significant variation in beta diversity. Boxes without stripe patterns are within gastrointestinal site/sex/age-class comparisons, and boxes with striped patterns black are between gastrointestinal site/sex/age-class comparisons.

In terms of beta diversity, there were significant effects from gastrointestinal tract site, age-class, and sex; however, the effects had very little explanatory power regarding variation in weighted UniFrac distances (gastrointestinal: R^2^ = 0.08, *p* = 0.03; Age-class: R^2^ = 0.03, *p* = 0.05; sex: R^2^ = 0.005, *p* = 0.007). Ordination plots show colon communities were more conserved. We also observed substantial overlap in microbiome composition between the two sites (Supplementary Figure S2); yet, there appeared to be no substantiated clusters on PCoA plots for gastrointestinal site or age-classes (Supplementary Figure S2). Indeed, jejunum and colon appeared to harbor phylogenetically similar GMBs, showing very little difference in intra- and inter-variation in either phylogenetic representation or relative abundance (Fig. 1A). Weighted UniFrac distances also showed little difference within and between sexes (Fig. 1B). For age-classes, we detected the greatest variation within adults (Fig. 1C). PERMDISP results were not significant for any of the three factors.

### Community composition of the gastrointestinal tract sites

**Figure 2:**
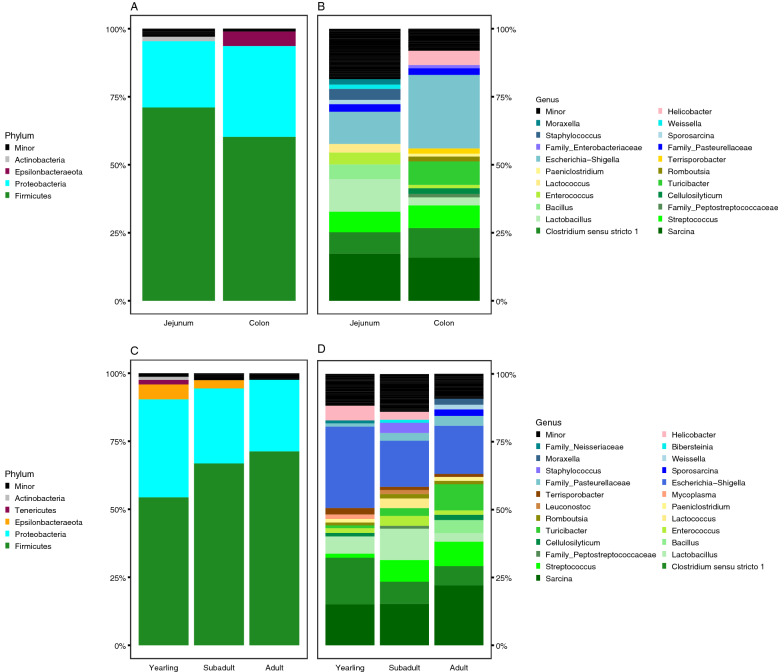
Bar charts displaying the relative abundance of sequences of the sampled black bears derived from major bacterial (left) phyla and (right) genera in each (**A**,**B**) gastrointestinal site and among (**C**,**D**) age-classes.

We identified three major phyla (Firmicutes, Proteobacteria, Actinobacteria) in the jejunum and three major phyla (Firmicutes, Proteobacteria, and Epsilonbacteraeota) in the colon (Fig. 2A). We defined major taxa as representing > 1% of amplicon sequence variants (ASVs). In addition to the three major phyla identified in each gastrointestinal tract site, we identified 21 minor phyla that were present at < 1% relative abundance. Firmicutes and Proteobacteria were the only major phyla detected in all samples, with Firmicutes being the most dominant phylum in both the jejunum community (71% ± 34% SD) and colon community (60% ± 33% SD), whereas Proteobacteria was the second most dominant phylum in the jejunum community (24% ± 30% SD) and the colon community (33% ± 30% SD). Actinobacteria (1.6% ± 2.9% SD) was a third phylum that was unique to the jejunum, while Epsilonbacteraeota (5.4% ± 10% SD) was a third phylum unique to the colon. All other phyla were minor in both gastrointestinal communities.

At the genus level, we identified 21 major genera across both gastrointestinal sites. The jejunum harbored 13 major taxa, six of which were unique to the jejunum. The colon harbored 15 major taxa, eight of which were found solely in the colon communities (Fig. 2B). *Sarcina* (17% ± 33% SD) dominated the jejunum, followed by *Lactobacillus* (12% ± 26% SD), and *Escherichia-Shigella* (12% ± 20% SD). *Escherichia-Shigella* dominated the colon community (27% ± 27% SD), followed by *Sarcina* (15.8% ± 28% SD).

### Community composition of age-classes

In yearlings, we identified five major phyla (Firmicutes, Proteobacteria, Epsilonbacteraeota, Actinobacteria, and Tenericutes). We found three major phyla in subadults (Firmicutes, Proteobacteria, and Epsilonbacteraeota), and two major phyla in adults (Firmicutes, and Proteobacteria; Fig. 2C). Similar to the colon and jejunum GMB communities, Firmicutes and Proteobacteria were the only major phyla detected in all age-classes. For yearlings, Firmicutes was the most dominant phylum (54.4% ± 31% SD), followed by Proteobacteria (36% ± 25% SD). The rest of the yearling GMB was composed of Epsilonbacteraeota (5.5% ± 14% SD), Actinobacteria (1.1% ± 2.6% SD), and Tenericutes (1.6% ± 5.8% SD); the latter was unique to yearlings. In subadults, Firmicutes (67% ± 32% SD) were the dominant phylum, followed by Proteobacteria (28% ± 28% SD) and Epsilonbacteraeota (3% ± 7% SD). The only dominant phyla in adult GMBs were Firmicutes (71% ± 37% SD) and Proteobacteria (26% ± 236% SD); all other phyla were minor (< 1% of ASVs).

At the genus level, we identified 25 major taxa across all three age-classes (Fig. 2D). Yearlings harbored 15 major taxa; however, only two taxa were unique to that age-class: *Mycoplasma* (1.6% ± 5.8% SD) and a genus of the family Neisseriaceae (1% ± 3.9% SD). All other taxa found in yearlings were also detected in subadults and/or adults. Further, although Actinobacteria and Tenericutes were major phyla in yearlings, no genera within these phyla constituted > 1% of the yearling GMB. Subadults harbored five unique taxa: *Staphylococcus* (3.8% ± 1.9% SD), *Lactococcus* (3.5% ± 1.2% SD), *Leuconostoc* (1.5% ± 4.4% SD), *Bibersteinia* (1.1% ± 3.9% SD), and an unclassified bacterium in the family Peprostreptococcacea (1.0% ± 2.2% SD). Subadults also had the largest number of minor taxa (*n* = 280). Adult black bears harbored four unique taxa: *Bacillus* (4.9% ± 2.1% SD), *Sporosarcina* (2.4% ± 1.1% SD), *Moraxella* (2.2% ± 7.6% SD), and *Weissella* (1.7% ± 6.9% SD). The GMBs of yearling and subadult black bears were dominated by *Escherichia-Shigella* (30% ± 23% SD; 17% ± 24% SD, respectively). The second most dominant genus found in yearling black bears was *Clostridium *sensu stricto* 1* (17% ± 26% SD), followed by *Sarcina* (15% ± 33% SD). *Sarcina* was the second most dominant genus in subadults (15% ± 30% SD), followed by *Lactobacillus* (12% ± 26% SD). *Sarcina* (22% ± 33% SD) dominated the GMB of adults, followed by *Escherichia-Shigella* (18% ± 29% SD), and *Turicibacter* (9.6% ± 23% SD).

We found a total of 23 ASVs that were differentially represented between the jejunum and colon at genus level (LDA score ≥ 3.65, *p* < 0.05; Table 3). In the jejunum, we identified seven differentially abundant bacteria. All were minor genera belonging to the three major phyla (4 Proteobacteria, 2 Actinobacteria, 1 Firmicutes; Table 3), though none could be classified below the level of order.

**Table 3:** Microbial taxa significantly (*p* < 0.05) enriched in black bear (*Ursus americanus*) jejunum versus colon, as determined by LEfSe analysis.

Phylum	Class	Order	Family	Genus	Log (LDA)
**Jejunum**					
Firmicutes	Bacilli	Bacillales	Unidentified	Unidentified	4.68
Proteobacteria	Gammaproteobacteria	Pseudomonadales	Unidentified	Unidentified	4.07
Proteobacteria	Alphaproteobacteria	Unidentified	Unidentified	Unidentified	4.05
Actinobacteria	Actinobacteria	Actinobacteria	Unidentified	Unidentified	3.9
Actinobacteria	Actinobacteria	Unidentified	Unidentified	Unidentified	3.85
Proteobacteria	Gammaproteobacteria	Betaproteobacteriales	Unidentified	Unidentified	3.84
Proteobacteria	Alphaproteobacteria	Rhizobiales	Unidentified	Unidentified	3.76
**Colon**					
Proteobacteria	Gammaproteobacteria	Enterobacteriales	Unidentified	Unidentified	4.86
Proteobacteria	Gammaproteobacteria	Enterobacteriales	Enterobacteriaceae	Unidentified	4.86
Proteobacteria	Gammaproteobacteria	Enterobacteriales	Enterobacteriaceae	*Escherichia-Shigella*	4.84
Proteobacteria	Alphaproteobacteria	Rhizobiales	Xanthobacteraceae	Unidentified	4.75
Firmicutes	Erysipelotrichia	Erysipelotrichales	Erysipelotrichaceae	*Turicibacter*	4.74
Proteobacteria	Gammaproteobacteria	Unidentified	Unidentified	Unidentified	4.74
Firmicutes	Erysipelotrichia	Erysipelotrichales	Erysipelotrichaceae	Unidentified	4.73
Firmicutes	Erysipelotrichia	Unidentified	Unidentified	Unidentified	4.72
Epsilonbacteraeota	Unidentified	Unidentified	Unidentified	Unidentified	4.46
Epsilonbacteraeota	Campylobacteria	Campylobacterales	Unidentified	Unidentified	4.46
Epsilonbacteraeota	Campylobacteria	Campylobacterales	Helicobacteraceae	*Helicobacter*	4.45
Epsilonbacteraeota	Campylobacteria	Campylobacterales	Helicobacteraceae	Unidentified	4.45
Firmicutes	Clostridia	Clostridiales	Clostridiaceae1	*Clostridium sensus tricto1*	4.43
Epsilonbacteraeota	Campylobacteria	Unidentified	Unidentified	Unidentified	4.43
Firmicutes	Clostridia	Clostridiales	Peptostreptococcaceae	Unidentified1	4.17
Firmicutes	Clostridia	Clostridiales	Peptostreptococcaceae	Unidentified2	3.65

**Figure 3:**
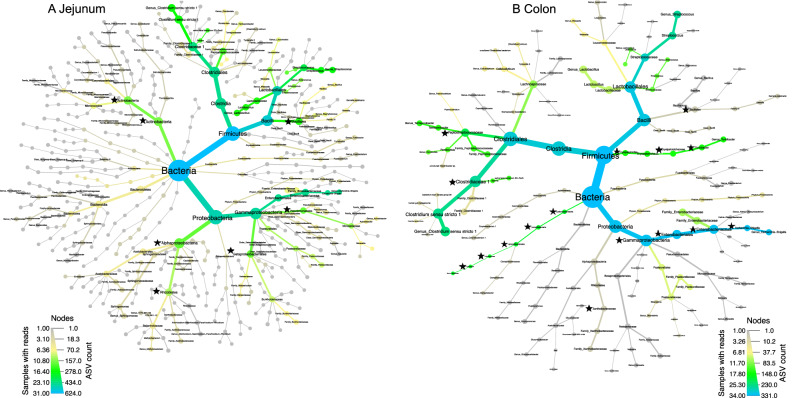
Phylogenetic heat trees representing the community structure as observed in (**A**) jejunum and (**B**) colon from rarefied sequencing data. Taxonomic hierarchy is based on ASV classification at to the genus level. Node width is proportional to the number of ASVs classified as that taxonomic level, and edge size is proportional to the number of reads. In the (**A**) jejunum, we observed a higher level of phylogenetic diversity, whereas the (**B**) colon hosted fewer phyla but more than twice the number of significantly and enriched taxa. Differentially significant taxa at each gastrointestinal site were detected via LEfSe and are indicated here by black stars.

We identified five unique and enriched major bacterial taxa in the colon: *Turicibacter* (8.6% ± 19% SD), *Helicobacter* (5.3% ± 10% SD), one unidentified genus within the family Enterobacteriaceae (1.2% ± 3% SD), and two unidentified genera in the family Peptostreptococcaceae (1.2% ± 1.9% SD; Table 3). Further, although found in both the jejunum and colon, *Escherichia-Shigella*, *Clostridium *sensu* stricto1,* and nine unidentified minor genera within the three major phyla (Firmicutes, Proteobacteria, and Epsilonbacteraeota) were significantly more abundant in the colon. Although colon samples harbored approximately twice as many enriched bacterial taxa as the jejunum, the majority of the differential colonic membership comprised unidentified genera within the same lineages occurring across the taxonomic hierarchy of the three major phyla (e.g., family, order, class). Conversely, we found a greater degree of phylogenetic variability among enriched bacteria in the jejunum compared to the colon (indicated by black stars in Fig. 3). Further, GMB taxonomic structure from the kingdom level to genus in the jejunum featured a greater abundance of minor taxa contributing to overall GMB composition, whereas the colon was dominated by fewer taxa primarily from the major phyla (Fig. 3). LEfSe results revealed no significantly enriched bacteria between age-classes.

## Discussion

To our knowledge, our research was the first to compare microbial communities in the jejunum and colon in a wild carnivore population. Here, we compared GMB structure between two operationally distinct regions of the gastrointestinal tract and among three age-classes of American black bears. Similar to previous studies on captive specimens^12^, black bear GMB membership and alpha diversity were influenced, in part, by age-class, with subadults’ GMBs harboring greater phylogenetic diversity compared to adults (Fig. 2). Similar to other bear species, the colonic GMB of wild American black bears was dominated by the phyla Firmicutes and Proteobacteria^40–45^.

Although weighted UniFrac distances were significantly different between gastrointestinal sites, sexes, and among age-classes, these factors appeared to be minor drivers of GMB composition. Weighted UniFrac PCoA plots also showed no distinct clustering or discernible patterns (Supplementary Figure S1). Given the different physiologies and micro-environments of the jejunum and colon, with fat, carbohydrates, and protein absorption occurring in the jejunum, and fermentation primarily occurring in the colon, the lack of discernible differences as measured in for distance matrices and alpha diversity was unexpected, especially considering that differences in community structure and membership have been observed previously across gastrointestinal sites in other omnivorous species^37^. However, perhaps due to site-specific environmental differences (i.e., host digestive processes and associated nutrient availability, we did observe that the colon contained high levels of differently enriched taxa from unknown but closely related lineages of bacteria (Table 3). Three differently enriched taxa in the colonic GMB community (*Turicibacter, Clostridium *sensu stricto* 1, and Escherichia/Shigella*) were also previously found to be major genera in captive Asiatic black bear^44^ and giant panda^38^ GMBs. By contrast, jejunum GMBs featured more minor taxa and phylogenetic branching contributing to jejunum GMB community composition (Figs. 2, 3).

Potential explanations for our findings could be that black bears’ generalist diet and rapid digestion time (13 h for meat/hair and 7 h for foliage)^28^, and the overall simplicity of the ursine gastrointestinal tract (i.e., short length and lack of vestigial organs such as appendix and cecum) may keep the jejunum and colon in a constant state of disturbance and thus prevent the development of discernible differences in community structure. Specifically, as generalist consumers with a simplistic gastrointestinal tract and rapid digestion, the climax community of the black bear GMB could be dominated by bacteria that function as pioneer species in other hosts with more complex guts and longer retention (i.e., succession) times. Although bacterial colonization is prompt, rapid transit time from consumption to defecation could facilitate constant and rapid turnover of microbial membership, preventing succession from progressing beyond a pioneer stage and favoring generalist/opportunistic microbial species. Furthermore, the black bear’s simple gastrointestinal tract affords no refuge where microbes can escape the constant turnover. The cecum and appendix are important morphological features whose function appear to be linked to immune system maintenance in mammals^46,47^, involving preservation of microbial biofilms in a location that is relatively sheltered from the main flow of intestinal contents^46^. This function is suggested to be important for the recovery of the microbiome following microbial loss due to diarrheal illnesses^46,48,49^ and as a reservoir for microbes^46^, a concept corroborated by recent studies^37,50^. For example, Greene and McKenney^50^ opportunistically sampled GMBs from the appendix, cecum, and colon of deceased captive aye-ayes and found that the appendix harbors distinct microbial diversity and composition, whereas the cecum and colonic communities were more homogenous^50^. Although species with physiologically more complex gastrointestinal tracts might harbor more stable microbial communities, carnivores such as black bears possess neither a cecum nor an appendix and thus lack a potential microbial reservoir to buffer microbial communities from the constant disturbance associated with a simple gastrointestinal tract and rapid food passage transit times.

The presumed turn-over of microbial communities within the gastrointestinal tract of black bears might serve as an evolutionary advantage, not in association with specialized digestion of specific foods, but regarding adiposity. In humans and mice, dysbiosis (i.e., an imbalance in microbial membership, often brought about through dietary shifts and often presumed to be detrimental) and low GMB diversity can lead to increased capacity for energy harvest and obesity^16,51,52^. Although obesity has been linked to health aliments in humans^53,54^, the need for black bears to undergo hyperphagia (i.e., physiological Stage IV) to rapidly gain weight is paramount for reproductive success and survival during torpor (i.e., physiological Stage I). For example, previous research shows that the seasonal composition and structure of GMBs in brown bears differ between physiological stages, with gut microbiota promoting energy storage during hyperphagia^40^. Thus, a constant state of microbial disturbance within black bear GIT could lead to more rapid weight gain.

Although we did not observe any differentially enriched bacteria among any age-classes, the overall observed increase in phylogenetic diversity could provide evidence of GMB colonization across the gastrointestinal tract as bear age. For instance, Faith’s PD was the lowest in yearlings, peaked in subadults, and subsequently decreased in adults (Fig. 1C). Notably, we also detected greater variability in Faith’s PD among subadults (Supplementary Figure S1), which could suggest subadults were in an intermediate succession states as previously observed in captive lemurs^12^. Additionally, we observed 5 major phyla, including greater proportions of *Helicobacter* and *Escherichia-Shigella*, both known potential pathogens, in yearlings (Fig. 1C,D). Both number of phyla and the relative abundance of *Helicobacter* gradually decrease in subsequent age-classes. Our results could suggest that as black bears mature, their bodies are better able to filter or select for specific microbes, i.e., via the immune system.

Interestingly, we also discovered the presence of the minor genus *Ursidibacter* (family Pasteurellaceae) in several jejunum and colon samples in the present study. *Ursidibacter* (family: Pasteurellaceae) was first sequenced from oral-cavity swabs collected from both wild and captive polar bears, and from captive brown bears^55^. Almost all members of the family Pasteurellaceae appear to be closely coupled to a single vertebrate host and are believed to be adapted to specific habitats^56^. Pasteurellaceae are generally pathogenetic or commensal bacteria, unable to survive in external environments, and have been found in the upper respiratory tract, throat, reproductive tract, and the gastrointestinal tract of vertebrate hosts^56^. To our knowledge, *Ursidibacter* has not been reported in other Ursid GMB communities since it was first sequenced in 2015, which may result from the range and bias of reference databases/versions used for bioinformatic analysis. Like the genus *Prevotella* in non-human primates^57,58^, *Ursidibacter* may have co-evolved and radiated with the family Ursidae.

With the increasing vulnerability of large carnivore populations to human-mediated environmental change worldwide, emphasis on bettering management and conserving these charismatic and ecologically significant species is paramount. Studying carnivore GMBs provides an opportunity to identify effects of anthropogenic pressures on carnivores not solely from a behavioral perspective or as a result of direct persecution but also in revealing how carnivores respond physiologically to human pressures (e.g., habitat degradation, access to processed/human foods) and the potential consequences of those physiological responses to the health of carnivores. Moreover, by better understanding the GMBs of generalist hosts and how the GMB responds to environmental change, wildlife managers can consider the value of incorporating strategies to promote holobiont conservation (e.g., the conservation of both host and GMB diversity)^59^ when striving to create effective management plans for species coping with human-mediated environmental change. To better understand the potential co-evolutionary history of Ursidae and their GMBs, future research could expand upon our study by investigating (1) whether dynamic, low-diversity GMBs may provide an evolutionary advantage for wildlife species with unique life histories such as black bears with future studies which incorporate multi-omics (i.e., metabolomic, metatranscriptomic) and (2) the bacterial lineage of *Ursidibacter*.

## Methods

### Study area and sample collection

We received an exemption from review by the Northern Michigan University’s Institutional Animal Care and Use Committee because samples were collected opportunistically from dead bears that were legally harvested by hunters, separate from this study. We collected samples with permission from individual hunters/guides under a Michigan DNR—Wildlife Division—Scientific Collector's Permit (#SC 1613).

We sampled black bears across the Upper Peninsula (UP) of Michigan, USA (7° 00′–45° 09′ N, 90° 18′–84° 37′ W). The UP consists of primarily coniferous and deciduous hardwood forests with intermittent conifer swamps, wetlands, shrub patches, and agriculture with elevation ranging between 170 to 600 m above sea level. Average daily temperatures during the September–October sampling frame varied from a low of − 1.6 °C to a high of 22 °C. We collected colon and jejunum content from legally harvested black bear and one roadkill bear (*n* = 35) within 30 min of death during the annual fall harvest season (September 10–October 26, 2018). Each guide/hunter was provided pre-packaged, color coordinated sampling kits. All kits were packed under sterile conditions in the lab, and hunters did not open the kits until after they had harvested the bear. Prior to collection, we met with each hunter/guide face to face individually to review the protocol as literature in citizen science have established face-to-face interactions are known to increase motivation and participation in online projects^60^. Further, Budde et al.^61^ demonstrated that a combination of technical instruction (i.e., provided kits with clearly worded and color-coded protocols/materials) and instruction (i.e., face-to-face meetings) combine synergistically to significantly reduce error rates.

After harvesting the bears, guides/hunters donned nitrile gloves and used the sterile tongue depressor to collect colon samples from the anus and immediately placed the sample into centrifuge tubes containing 7 mL of 95% ethanol. After guides/hunters field dressed the bears by opening the abdomen and mobilizing the stomach and intact intestines, they donned new sterile nitrile gloves to prevent contamination of samples with human/environmental microbes, used the provided 16-inch string to sample the gut microbiome at a standardized distance from the pyloric sphincter and used a clean knife to make a small incision in the jejunum. Finally, squeezed the intestine to extrude gut content straight into a pre-labelled sample tube containing 7 mL of 95% ethanol (see Supplementary Figure S3 for hunter instructions). All samples were stored at room temperature until microbial DNA was extracted (~ 50 days). Sex was recorded and the Michigan Department of Natural Resources provided age data from teeth they collected per their harvest registration protocols (Supplementary Table S3). Using this data, bears were categorized into age-classes (i.e., yearling = 1, subadult = 2–3, adult ≥ 4).

### DNA isolation and sequencing

A total of 66 samples were collected from black bears across all age-classes to be used for 16S sequencing from seven yearlings (all yearlings had both jejunum and colon samples), 15 subadults (13 subadults had both jejunum and colon samples), 11 adults (nine adults had both colon and jejunum samples), and two bears from unknown age-classes (both with jejunum and colon samples; metadata for all individuals are available in Supplementary Table S3). We extracted microbial DNA from jejunum and colon samples using DNeasy PowerSoil Kits (QIAGEN, Hilden, Germany), following the manufacturer’s protocol with the addition of (1) a heat-step of 10 min at 65 °C at the beginning of the protocol to breakdown proteins and (2) a second elution as the final step of extraction, as previously described by McKenney et al.^62^. We quantified DNA yields using a NanoDrop 2000c (ThermoFischer Scientific, Massachusetts, USA) and stored extractions at − 20 °C. After all extractions were complete, standardized DNA aliquots were shipped to Argonne National Laboratory (Lemont, IL, USA) for PCR amplification and paired-end DNA sequencing of the 16S rRNA V4 gene region, according to methods described by Caporaso et al.^63^.

### Bioinformatic analysis

Multiplexed EMP-paired-end sequence reads were imported into Quantitative Insights Into Microbial Ecology (QIIME2, version 2019.4)^64^ and demultiplexed. Sequences were joined, denoised, filtered to remove chimeras and residual Phix reads, and dereplicated; ASVs were called using the DADA2 QIIME2 plugin^65^, and sequence lengths were truncated to 150 bp.

### Taxonomic classification

We used the SILVA 99 database for V4 region (version 132)^66^ to assign taxonomic classification in QIIME2, using a trained Naïve Bayes sklearn classifier^67^ to classify organisms at the genus level. Sequences were aligned with MAFFT^68^, a plugin for phylogenetic diversity analysis, which removes highly variable positions in the process. We further filtered samples to remove chloroplast, mitochondrial, and Archaea sequences, as well as unidentified microbial DNA unidentified below kingdom.

### Statistical analysis

All statistical analyses and visualizations were performed using R (version 3.6.1)^69^ and Rstudio (version 1.2.1335)^70^. We investigated the alpha diversity of GMB communities per gastrointestinal tract site through analysis of the Shannon and inverse Simpson diversity indices, and of Faith’s PD. We use linear mixed effects models (LMM) for analyses to determine the relationship between Faith’s PD and gastrointestinal tract sites. We included gastrointestinal tract site, sex and age-class (yearling = 1, subadult = 2–3, adult ≥ 4) as categorical fixed effects; alpha diversity indices as the response variables; and individual as a random effect. In each model, we checked residuals to confirm model requirements (e.g., normality, homoscedasticity, residuals). To determine whether interactions between main effects should be considered, we fit models with the maximum likelihood (ML) and compared likelihood ratio tests, which performs Wald Chi-squared tests for LMM. Final models were fit with restricted maximum likelihood (REML). We also determined the significance of main effects and interactions within the top models using Wald Chi-squared tests. Faith’s PD and inverse Simpson diversity values were log-transformed prior to analysis, to accommodate their skewed values. If main effects or interactions were significant, we acquired estimated marginal means (EMMs) of pairwise comparisons for post hoc testing with Tukey adjustment.

For beta diversity, we quantified compositional dissimilarity between gastrointestinal tract sites, sex, and age-class using the quantitative phylogenetic weighted UniFrac distance and plotted these data on principle coordination analysis (PCoA) ordination plots. We performed permutational multivariate analysis of variance (perMANOVA)^71^ on each distance matrix with a strata for subject ID. We implemented an analysis of multivariate homogeneity (PERMDISP)^72^, an analogue of Levene’s test for homogeneity of variance, again with a strata for subject ID, to test for significant differences in sample heterogeneity between gastrointestinal tract sites. Finally, we used Linear discrimination analysis Effect Size (LEfSe) in the Galaxy online tool (https://huttenhower.sph.harvard.edu/galaxy) to identify any ASVs that were differentially represented between gastrointestinal tract sites. We designated a logarithmic Linear Discriminate Analysis (LDA) score of 2.0 as the cut-off for LEfSe analysis^73^. We considered a *p *value threshold of 0.05 significant for each test performed. See Zenodo Digital Repository https://doi.org/10.5281/zenodo.4060480 for QIIME2 pipeline and code for statistical analysis.

## Supplementary information

Supplementary Information.

## Data Availability

The data and coding reported in this paper are available and will be archived in the Zenodo Digital Repository https://doi.org/10.5281/zenodo.4060480.
