# Supplementary material for: Wild black bears harbor simple gut microbial communities with little difference between the jejunum and colon
Source: Sci Rep. 2020 Nov 27;10:20779. doi: 10.1038/s41598-020-77282-w (PMC7695734; doi:10.1038/s41598-020-77282-w)
Supplement: Supplementary file 1 — Supplementary Information. [file 41598_2020_77282_MOESM1_ESM.pdf]

Wild black bears harbor simple gut microbial communities with little difference between the jejunum and colon

Sierra J. Gillman, Erin A. McKenney, Diana J. R. Lafferty

Supplementary Figure S1

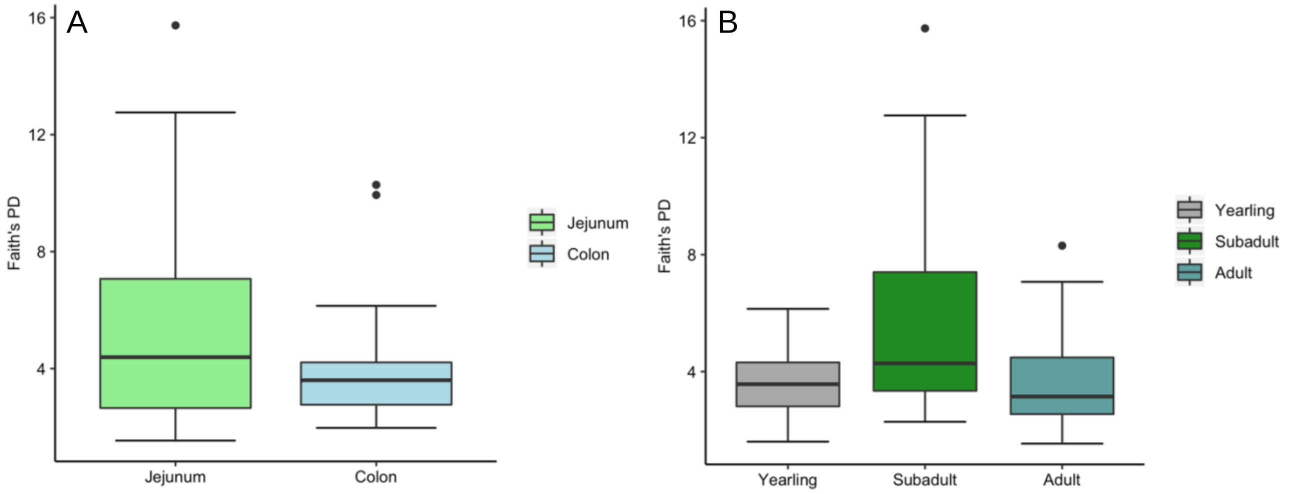

Supplementary Figure S1 | Box plots with standard deviations for Faith's PD for each **(A)** gastrointestinal site and **(B)** age-class. Gastrointestinal sites did not significantly differ in their degree of phylogenetic diversity. Age-class was a significant predictor for Faith's PD, and estimated marginal means revealed the significance difference was between subadults (3.74 ± 1.80) and adults (5.61 ± 3.42; p=0.05).

Supplementary Figure S2

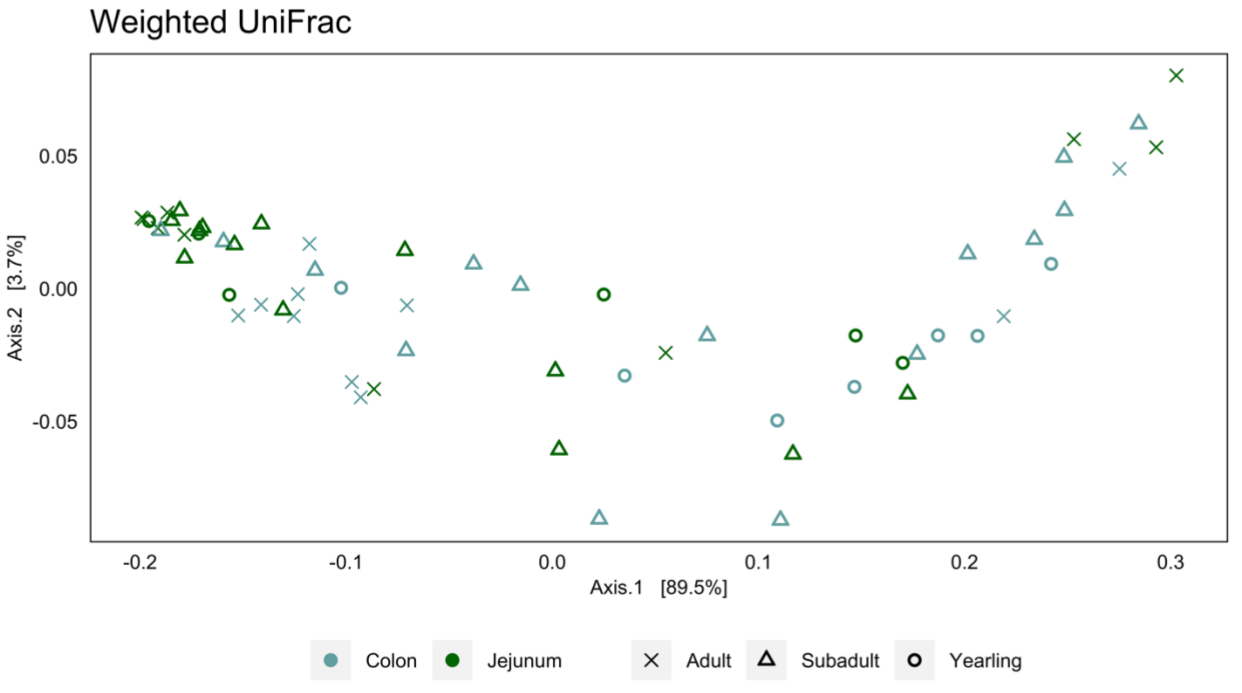

Supplementary Figure S2 | GIT site and age-class exhibit distinct microbiota communities. PCoA plots of weighted UniFrac distances of jejunum (green) and colon (blue). perMANOVA results for weighted UniFrac showed significant difference between GIT sites (R^2=0.07, p=0.03), sex (R^2=0.005, p=0.007), and age-class (R^2=0.04, p=0.001). On the PCoA plot, each symbol represents one GMB.

Supplementary Figure S3

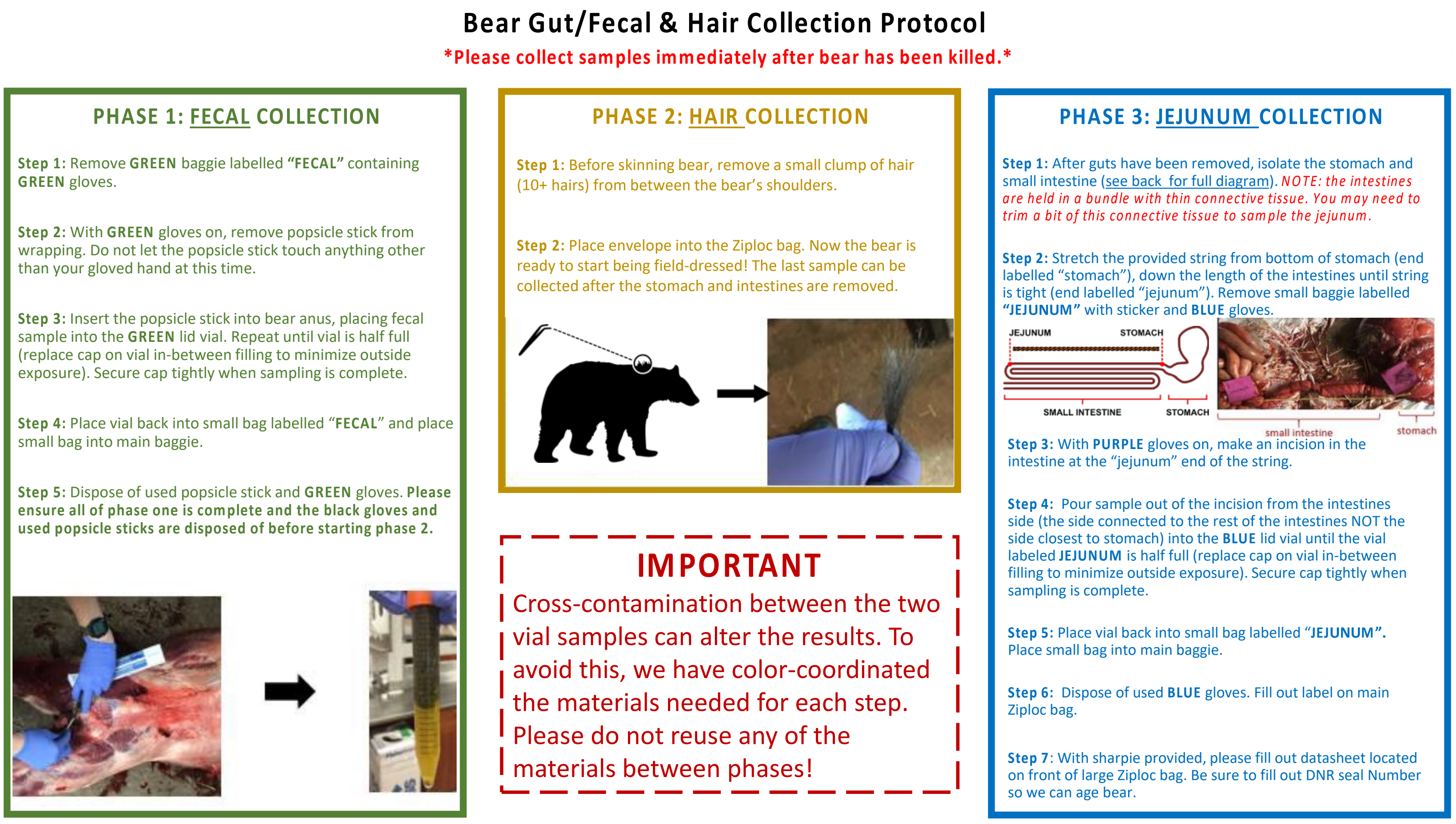

Supplementary Figure S3 | Sample collection instructions for hunters and guides who gave biological samples from black bears legally harvested during the Michigan 2018 black bear harvest. Samples were all collected within 30 minutes of death.

Supplementary Table S1 | Wald χ^2 tests for Alpha diversity model selection.

**A.** Faith's PD								
**Models:**								
model1	Log(PD)~GIT+Sex+AgeClass+(1|Subject)							
model2	Log(PD)~GIT+GIT×Sex+Sex+AgeClass+(1|Subject)							
model3	Log(PD)~GIT×AgeClass+Sex+GIT+AgeClass+(1|Subject)							
model4	Log(PD)~GIT+Sex+AgeClass+GIT×AgeClass+GIT×Sex+(1|Subject)							
**Models:**	**Df**	**AIC**	**BIC**	**logLik**	**deviance**	**χ^2 Chi**	**Df**	**Pr(>Chisq)**
model1	7	91.35	106.13	-38.68	77.35			
model2	8	92.90	109.78	-38.45	76.90	0.46	1	0.50
model3	9	93.44	112.44	-37.72	75.44	1.45	1	0.23
model4	10	95.31	116.41	-37.65	75.31	0.14	1	0.71
**B.** Shannon diversity								
**Models:**								
model1	Shannon~GIT+Sex+AgeClass+(1|Subject)							
model2	Shannon~GIT+GIT×Sex+Sex+AgeClass+(1|Subject)							
model3	Shannon~GIT×AgeClass+Sex+GIT+AgeClass+(1|Subject)							
model4	Shannon~GIT+Sex+AgeClass+GIT×AgeClass+GIT×Sex+(1|Subject)							
**Models:**	**Df**	**AIC**	**BIC**	**logLik**	**deviance**	**χ^2Chi**	**Df**	**Pr(>Chisq)**
model1	7	171.75	186.53	-78.88	157.75			
model2	8	173.61	190.50	-78.81	157.61	0.14	1	0.71
model3	9	174.11	193.10	-78.05	156.11	1.51	1	0.22
model4	10	176.07	197.18	-78.03	156.07	0.04	1	0.84
**A.** inverse Simpson diversity								
**Models:**								
model1	log(inverse Simpson)~GIT+Sex+AgeClass+(1|Subject)							
model2	log(inverse Simpson)~GIT+GIT×Sex+Sex+AgeClass+(1|Subject)							
model3	log(inverse Simpson)~GIT×AgeClass+Sex+GIT+AgeClass+(1|Subject)							
model4	log(inverse Simpson)~GIT+Sex+AgeClass+GIT×AgeClass+GIT×Sex+(1|Subject)							
**Models:**	**Df**	**AIC**	**BIC**	**logLik**	**deviance**	**χ^2 Chi**	**Df**	**Pr(>Chisq)**
model1	7	150.19	164.97	-68.10	136.19			
model2	8	151.85	168.74	-67.92	135.85	0.35	1	0.56
model3	9	153.14	172.14	-67.57	135.14	0.71	1	0.40
model4	10	154.94	176.05	-67.47	134.94	0.20	1	0.65

Supplementary Table S2 | Mean ± SD values for alpha diversity between gastrointestinal sites and among three age-classes for American black bear (*Ursus americanus).* For age-class, two black bears with unassigned age-classes and were removed from age-class analysis.

Group	Sample size (n)	Faith's PD	Shannon	Inverse Simpson
**A. Gut microbiome alpha diversity for two gastrointestinal sites**				
Jejunum	31	5.34 ± 3.34	1.64 ± 1.09	5.28 ± 7.08
Colon	34	3.99 ± 1.84	1.72 ± 0.67	3.77 ± 2.13
**B. Gut microbiome alpha diversity for three age-classes**				
Yearling	13	3.79 ± 1.31	1.66 ± 0.80	3.75 ± 2.25
Subadult	28	5.61 ± 3.42	1.79 ± 0.98	5.50 ± 7.38
Adult	20	3.74 ± 1.80	1.47 ± 0.86	3.53 ± 2.18
**C. Gut microbiome alpha diversity between two sexes**				
Female	27	5.15 ± 3.58	1.75 ± 1.06	5.90 ± 7.48
Male	38	4.27 ± 1.88	1.64 ± 0.75	3.71 ± 2.21

Supplementary Table S3 | Sex (females *n*= 14; males *n*= 21), age/age-class, and gastrointestinal site collection for each black bear (*Ursus americanus*) sampled during the 2018 the Upper Peninsula of Michigan black bear hunting season.

Bear	GIT	Sex	Age	Age-Class	Age=Class Sample Size (*n*)
B1	Colon & Jejunum	Male	1	Yearling	
B2	Colon & Jejunum	Female	1	Yearling	
B3	Colon & Jejunum	Male	1	Yearling	
B4	Colon & Jejunum	Male	1	Yearling	*n*= 7
B5	Colon & Jejunum	Male	1	Yearling	
B6	Colon & Jejunum	Male	1	Yearling	
B7	Colon & Jejunum	Male	1	Yearling	
B8	Colon & Jejunum	Male	2	Subadult	
B9	Colon & Jejunum	Female	2	Subadult	
B10	Colon & Jejunum	Male	2	Subadult	
B11	Colon & Jejunum	Female	2	Subadult	
B12	Colon	Male	2	Subadult	
B13	Colon & Jejunum	Female	2	Subadult	
B14	Colon & Jejunum	Female	2	Subadult	
B15	Colon & Jejunum	Male	2	Subadult	*n*= 15
B16	Colon & Jejunum	Male	3	Subadult	
B17	Colon	Female	2	Subadult	
B18	Colon & Jejunum	Male	3	Subadult	
B19	Colon & Jejunum	Male	2	Subadult	
B20	Colon & Jejunum	Male	3	Subadult	
B21	Colon & Jejunum	Male	2	Subadult	
B22	Colon & Jejunum	Female	2	Subadult	
B23	Colon & Jejunum	Female	13	Adult	
B24	Colon & Jejunum	Female	10	Adult	
B25	Colon & Jejunum	Male	6	Adult	
B26	Colon & Jejunum	Male	4	Adult	
B27	Colon & Jejunum	Female	7	Adult	
B28	Colon & Jejunum	Male	6	Adult	*n*= 11
B29	Colon & Jejunum	Female	5	Adult	
B30	Colon & Jejunum	Male	4	Adult	
B31	Colon	Male	5	Adult	
B32	Colon & Jejunum	Male	4	Adult	
B33	Jejunum	Female	8	Adult	
B34	Colon & Jejunum	Female	NA	Unknown	*n*= 2
B35	Colon & Jejunum	Female	NA	Unknown	
